# Intracellular Fate and Impact on Gene Expression of Doxorubicin/Cyclodextrin-Graphene Nanomaterials at Sub-Toxic Concentration

**DOI:** 10.3390/ijms21144891

**Published:** 2020-07-10

**Authors:** Daniela Caccamo, Monica Currò, Riccardo Ientile, Elisabetta AM Verderio, Angela Scala, Antonino Mazzaglia, Rosamaria Pennisi, Maria Musarra-Pizzo, Roberto Zagami, Giulia Neri, Consolato Rosmini, Monica Potara, Monica Focsan, Simion Astilean, Anna Piperno, Maria Teresa Sciortino

**Affiliations:** 1 Department of Biomedical Sciences, Dental Sciences and Morpho-Functional Imaging, Polyclinic Hospital University, 98125 Messina, Italy; dcaccamo@unime.it (D.C.); moncurro@unime.it (M.C.); ientile@unime.it (R.I.); 2 School of Science and Technology, Centre for Health, Ageing and Understanding of Disease, Nottingham Trent University, Nottingham NG11 8NS, UK; elisabetta.verderio-edwards@ntu.ac.uk; 3 Department of Chemical, Biological, Pharmaceutical and Environmental Sciences, University of Messina, V.le F. Stagno d’Alcontres 31, 98166 Messina, Italy; ascala@unime.it (A.S.); rpennisi@unime.it (R.P.); mmusarrapizzo@unime.it (M.M.P.); giulia.neri@unime.it (G.N.); conso.rosmini@gmail.com (C.R.); 4 CNR-Istituto per lo Studio dei Materiali Nanostrutturati (CNR-ISMN), Department of Chemical, Biological, Pharmaceutical and Environmental Sciences, University of Messina, V.le F. Stagno d’Alcontres 31, 98166 Messina, Italy; antonino.mazzaglia@cnr.it (A.M.); roberto.zagami@ismn.cnr.it (R.Z.); 5 Department of Innate Immunology, Shenzhen International Institute for Biomedical Research, 140 Jinye Ave, Building A10, Life Science Park, Dapeng New District, Shenzhen 518119, China; 6 Nanobiophotonics and Laser Microspectroscopy Center, Interdisciplinary Research Institute in Bio-Nano-Sciences, Babes-Bolyai University, T. Laurian Str. 42, 400271 Cluj-Napoca, Romania; monica.potara@phys.ubbcluj.ro (M.P.); monica.iosin@phys.ubbcluj.ro (M.F.); simion.astilean@phys.ubbcluj.ro (S.A.); 7 Department of Biomolecular Physics, Faculty of Physics, Babes-Bolyai University, M Kogalniceanu Str. 1, 400084 Cluj-Napoca, Romania

**Keywords:** nanostring^®^, graphene, cyclodextrin, doxorubicin, gene expression, FLIM, Raman mapping, nanomaterials

## Abstract

The graphene road in nanomedicine still seems very long and winding because the current knowledge about graphene/cell interactions and the safety issues are not yet sufficiently clarified. Specifically, the impact of graphene exposure on gene expression is a largely unexplored concern. Herein, we investigated the intracellular fate of graphene (G) decorated with cyclodextrins (CD) and loaded with doxorubicin (DOX) and the modulation of genes involved in cancer-associated canonical pathways. Intracellular fate of GCD@DOX, tracked by FLIM, Raman mapping and fluorescence microscopy, evidenced the efficient cellular uptake of GCD@DOX and the presence of DOX in the nucleus, without graphene carrier. The NanoString nCounter™ platform provided evidence for 34 (out of 700) differentially expressed cancer-related genes in HEp-2 cells treated with GCD@DOX (25 µg/mL) compared with untreated cells. Cells treated with GCD alone (25 µg/mL) showed modification for 16 genes. Overall, 14 common genes were differentially expressed in both GCD and GCD@DOX treated cells and 4 of these genes with an opposite trend. The modification of cancer related genes also at sub-cytotoxic G concentration should be taken in consideration for the rational design of safe and effective G-based drug/gene delivery systems. The reliable advantages provided by NanoString^®^ technology, such as sensibility and the direct RNA measurements, could be the cornerstone in this field.

## 1. Introduction

In the last years, graphene-based materials (G) due to their outstanding physicochemical properties have been proposed for promising biomedical/pharmaceutical applications, such as bio-imaging, drug/gene delivery, and biomolecular detection [1,2,3]. Numerous studies have been dedicated to the development and the biological response of G-based nanocarriers loaded with Doxorubicin (DOX), an effective chemotherapeutic agent used for the treatment of different kind of cancer [4,5,6]. Generally, literature data demonstrated superior therapeutic performance in cancer chemotherapy experiments, both in vitro and in vivo, due to the improved tissue penetration and cellular uptake of G-based nanocarriers [7,8]. However, the road of G in nanomedicine seems still very long and winding because the current knowledge about the G/cell interactions and the safety issues are not yet sufficiently clarified [9,10].

Pristine graphene (i.e., graphene in its original, unmodified form) has many remarkable properties but it is unable to be dispersed in water, making exploitation of its unique features challenging and less explored compared to its derivatives, that are more dispersible in aqueous medium, stable in physiological conditions, and further tunable for a wide range of applications, due to host functional groups [11,12]. G can differ on the basis of (i) synthetic approaches adopted for their preparation; (ii) not being homogeneous nature (several oxidation states, different lateral sizes and number of layers, colloidal stability, etc.); (iii) presence of impurities; (iv) nanocomposites obtained by combination with organic or inorganic compounds. All these G have been clamped into the umbrella term of “graphene” in the literature. The lack of univocal classification based on standardized characterization methods, as well the wide range of assays and cell lines used for its biological evaluation, are the main causes of the controversial literature reports about its safety issues [13].

In our ongoing program, aimed to the discovery of new G-based drug delivery systems [14,15,16], we expand the aims of our work focusing our attention on the modulation of gene expression induced by G-cell interaction. Differently from other nanomaterials, such as silica nanoparticles [17], the impact of G exposure in gene expression is a concern largely unexplored, till now [18,19,20,21].

**Figure 1:**
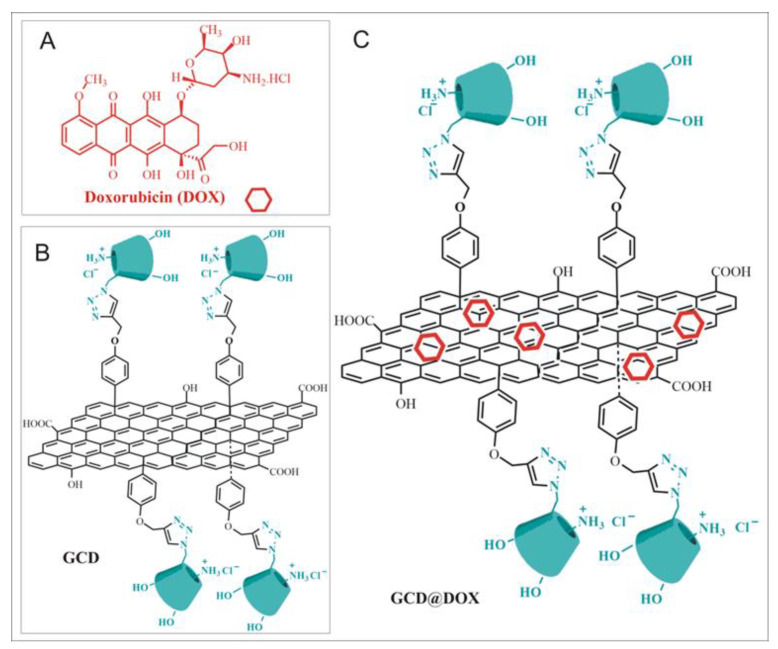
(**A**) Doxorubicin (DOX); (**B**) Schematic sketch of Graphene functionalized with Cationic Cyclodextrins (GCD platform); (**C**) Schematic sketch of DOX loaded on GCD (GCD@DOX).

Herein, we focus our attention on the effects of exposure of human epithelial type 2 (HEp-2) cells to GCD and GCD@DOX platforms (Figure 1). GCD is a functional graphene material, recently synthesized in our lab [16] belonging to the “graphene-family materials and cyclodextrins” [22], which was proposed as drug and/or gene carrier, due to its ability to avoid the entrapment in the acidic lysosomes (characteristic of the clathrin-mediated route), versus the efficient caveolae-mediated route [16]. GCD cellular uptake occurred via a specific caveolae-mediated endocytosis mechanism inhibited by genistein (tyrosine-kinase inhibitor that affects caveolae dynamics) and unaffected by filipin (inhibitor that sequesters cholesterol in the membrane and disrupts the lipid rafts) [16].

In this work, we used GCD to prepare GCD@DOX complex. Moreover, the changes in gene expression involved in molecular pathways regulating cell cycle progression were investigated for both GCD and GCD@DOX platforms. The physico-chemical properties of GCD and GCD@DOX were investigated by Dynamic Light Scattering (DLS) and Zeta-potential measurements and Raman spectroscopy. The intracellular trafficking of GCD@DOX was elucidated combining FLIM, Raman imaging and fluorescent microscopy outcomes. Finally, the abilities of GCD and GCD@DOX to induce changes in the expression of genes involved in processes associated to angiogenesis, extracellular matrix (ECM) modification and tumor metastasis were evaluated using NanoString nCounter™ platform, a novel digital molecular barcoding technology.

## 2. Results

### 2.1. Preparation and Characterization of GCD@DOX Complex

Graphene modified with cationic cyclodextrins (GCD) was prepared according to our previously reported procedure [16]. DOX was loaded on GCD with high entrapment efficiency by mixing aqueous GCD dispersion and DOX solution under ultrasonication. GCD@DOX complex was characterized by spectroscopy, such as DLS, ζ-potential measurements and Raman. Overall properties of GCD and GCD@DOX were reported in Table 1.

**Figure 2:**
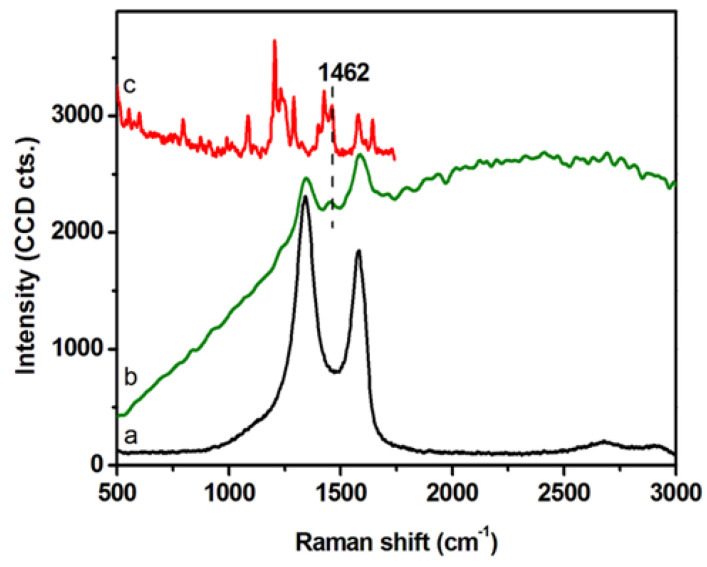
Raman spectra of GCD (**a**), GCD@DOX (**b**) on dried droplets collected using an excitation line at 532 nm. Raman spectrum of solid DOX (**c**) recorded using the 785 nm excitation line.

DLS and ζ-potential measurements were exploited to investigate the colloidal stability of GCD@DOX complex (Table 1). DLS analyses detected GCD and GCD@DOX as pseudo spherical large aggregates (D_H_ ~1 μm) and no size changes were observed along the time. DLS values were not indicative of the morphology and size of the nanoplatform, but they assessed the average hydrodynamic size of large graphene sheet aggregates in diluted aqueous dispersion [23]. TEM micrographs showed graphene layers of about 400 nm with an average thickness of 10–12 nm [16]. DLS measurements indicated similar sizes between GCD and GCD@DOX aggregates; whereas, a decrease of the ζ-potential value was detected for GCD@DOX (GCD about −9.5 mV vs. GCD@DOX about −16.6 mV). These findings indicated a higher colloidal stability of GCD@DOX complex in water compared to free GCD. The formation of GCD@DOX complex was further investigated by Raman spectroscopy (Figure 2). The Raman spectrum of GCD (spectrum (a) showed the characteristic G (~1584 cm^−1^) and D (~1348 cm^−1^) graphene bands [24]; whereas, a more complex Raman profile was observed for GCD@DOX (Figure 2, spectrum (b). Specifically, besides the typical G and D bands of graphene, a new weak band was evident at 1457 cm^−1^. This band was connected to that present at 1462 cm^−1^ (skeletal ring vibrations) in the Raman spectrum of DOX (Figure 2, spectrum (c) [25]. Moreover, the Raman vibrational bands of GCD and DOX overlapped with a broad band centered at around 2575 cm^−1^ (around 616 nm) attributed to fluorescence emission of DOX. Taken together, these spectral differences clearly proved the interaction between DOX and GCD due to the formation of GCD@DOX complex.

### 2.2. Intracellular Trafficking of GCD@DOX by FLIM and Raman Mapping and Fluorescent Microscopy

**Figure 3:**
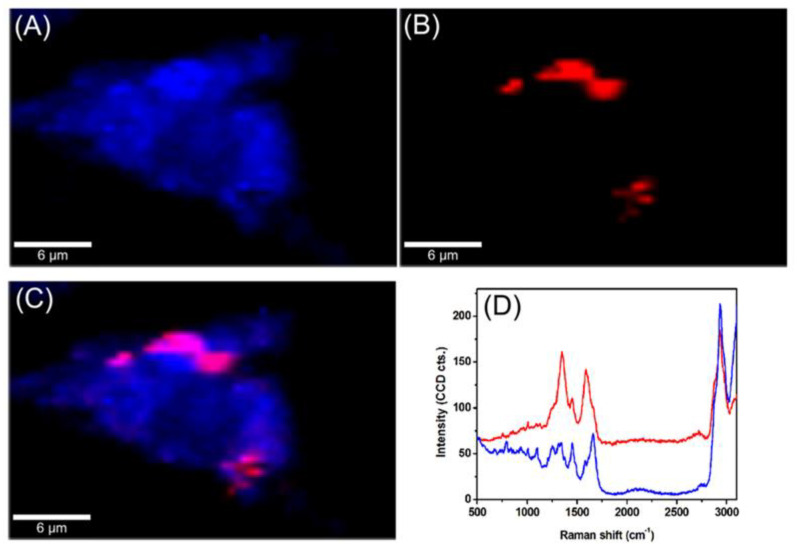
Raman maps of a single C26 colon carcinoma cells incubated for 24 h with GCD@DOX. (**A**) The map was obtained by plotting the intensity of C-H stretching vibrational bands of lipids at 2800–3100 cm^−1^ (**B**) The map was generated by plotting the intensity of the G band of graphene at 1584 cm^−1^. (**C**) Overlaid Raman maps presented in (**A**,**B**). (**D**) Extracted spectra corresponding to colored areas.

The intracellular trafficking of GCD@DOX was investigated by FLIM and Raman mapping using C26 colon carcinoma cells model. Owing to the intrinsic Raman signal of G nanoflakes, we employed confocal Raman microscopy to screen the uptake and localization of GCD@DOX inside live cells. This technique is a non-invasive analytical tool based on inelastic scattering of light by the molecules in a sample. A Raman spectrum provides information about the molecular composition, molecular structures, and molecular interactions in a sample. By scanning XY-plane areas of a sample at different z-depths and representing the intensity of selected Raman bands, one can construct a false-colored Raman map, which allows both the visual detection of the nanoagents inside cells and the identification of the biochemical profile of cellular constituents. The Raman map of a single C26 cancer cell was obtained by plotting the intensity of C-H stretching vibrational bands (2800–3100 cm^−1^) of lipids (Figure 3A). The visualization of GCD@DOX into C26 cancer cell was achieved by representing the intensity of the specific G band of graphene at 1584 cm^−1^ (Figure 3B). The overlaid Raman maps (Figure 3C) enabled the visual discrimination among nucleus, cytoplasmatic region and GCD@DOX. In particular, GCD@DOX can be seen as red spots inside the cytoplasm and around nucleus, suggesting that the GCD@DOX probably released DOX surrounding to the nucleus. The extracted Raman spectra corresponding to colored areas (Figure 3D) revealed obvious biochemical differences between red and blue areas. For instance, the Raman profile of the blue spectrum collected from the nucleus was dominated by the vibrational bands of cellular constituents at 2800–3100 cm^−1^ (lipids—stretching CH_2_), 1665 cm^−1^ (amide I—stretching CO coupled with deformation NH), 1453 cm^−1^ (lipids and proteins—deformation CH_2_, CH_3_), 1008 cm^−1^ (phenylalanine—ring breathing) and 787 cm^−1^ (ring breathing DNA/RNA bases). In contrast, the red spectrum presented a hybrid Raman profile containing the spectral signal of graphene, cellular constituents and fluorescence emission of DOX.

**Figure 4:**
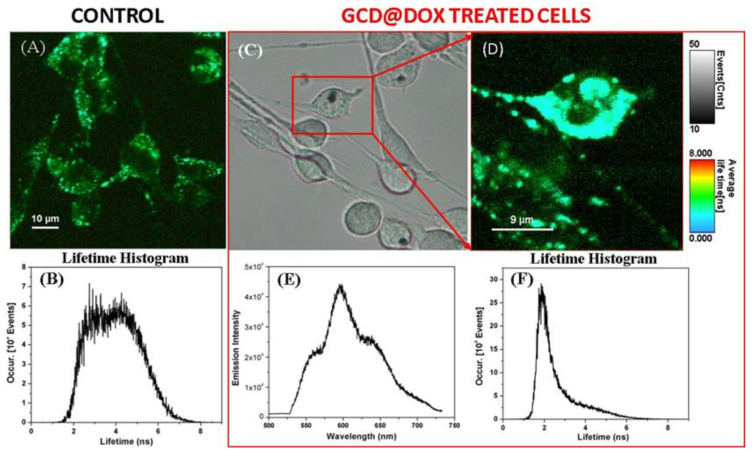
FLIM image and its corresponding lifetime histogram (**A**,**B**) of the untreated C26 colon cancer cells -as control, in comparison with the bright field (**C**) and FLIM image of the GCD@DOX treated cells (red box) for 24 h of incubation (**D**), its corresponding lifetime histogram (**F**), and the recorded emission spectrum (**E**) inside the marked cell in (**C**).

To confirm the cellular uptake of GCD@DOX, we invoked fluorescence lifetime imaging microscopy (FLIM), an ideal technique for live-cell imaging, which is able to display the spatial distribution of exited state lifetimes of a fluorophore, its molecule’s chemical microenvironment or different binding state. Specifically, Figure 4A shows the FLIM image of the control C26 cancer cells, without therapeutic agent. A slight autofluorescence signal emitted by the control cells can be observed with an average lifetime value of about 4 ns (Figure 4B), due to the diversity of endogenous fluorophores and flavin coenzymes [26]. FLIM image in Figure 4D showed successfully internalization and cellular distribution of the GCD@DOX inside C26 colon cells after 24 h of incubation having a shorter lifetimes value of 2.1 ns. Furthermore, by using the Andor spectrograph coupled to our Microtime 200 inverted FLIM microscope, we were also able to collect the emission spectrum of the DOX loaded onto the GCD inside the marked C26 colon cells from the bright-field microscopic image (Figure 4E). The emission located at around 600 nm upon diode laser excitation at 485 nm (Figure 4E), confirming once again the successful cellular internalization of GCD@DOX. Bright field image was shown in Figure 4C.

Finally, the intracellular fate of loaded and/or released DOX was visualized by confocal microscopy images of HEp-2 cells exposed to GCD@DOX (25 μg/mL) for 24 h.

**Figure 5:**
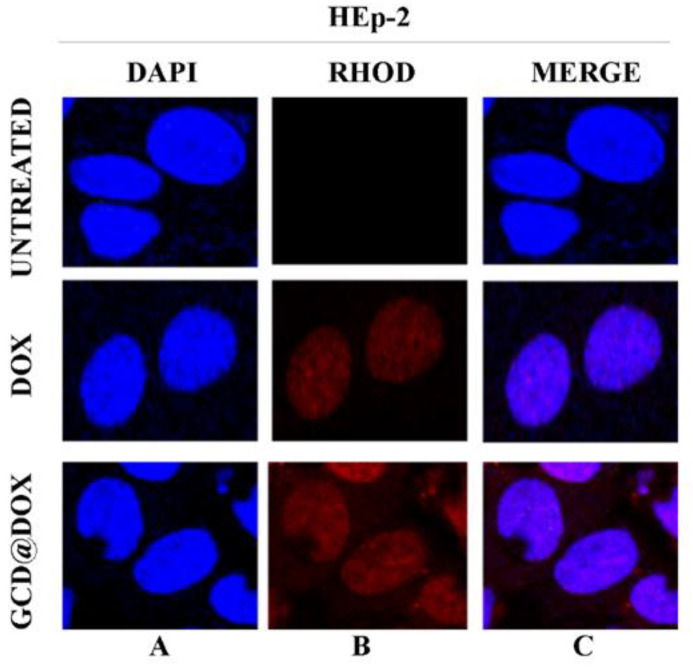
In vitro study of cellular uptake of GCD@DOX in HEp-2 cells. The HEp-2 cells were untreated or treated with GCD@DOX (25 μg/mL) and DOX (1.25 μg/mL). After 24 h the cells were harvested and the autofluorescence of GCD@DOX and DOX was evaluated through fluorescence analysis. A drop of mounting solution (ProLong™ Diamond Antifade Mountant with DAPI-Invitrogen p36971) was used for 30 min in a dark room for confocal microscopy analysis. Standard /DAPI/FITC/TRITC filters were used to detect the intracellular fluorescence. The column (**A**) represents untreated and treated cells with DOX and GCD@DOX and visualized with DAPI filter. The column (**B**) represents untreated and treated cells with DOX and GCD@DOX and visualized by Rhodamine filter. The (**C**) column represents the merged images. The images were captured and processed using Confocal laser scanning microscopy Leica TCS SP8 (Magnification, 63×).

Figure 5 represented HEp-2 cells untreated and treated with DOX and with GCD@DOX, visualized with DAPI filter (column A) to detect the nuclei, or visualized by Rhodamine filter (column B) to visualize the natural red color of free and entrapped DOX. Column C represented the merged images.

The DAPI-Rhodamine overlapped images (column C) showed the perfect match between the emitted red fluorescence of free DOX and GCD@DOX and the nuclei position (Figure 5). The patterns of cell distribution of DOX was visibly widespread in nuclear compartmentation, accordingly to doxorubicin-chromatin engagement reported in literature [27].

### 2.3. Cytotoxicity Study

**Figure 6:**
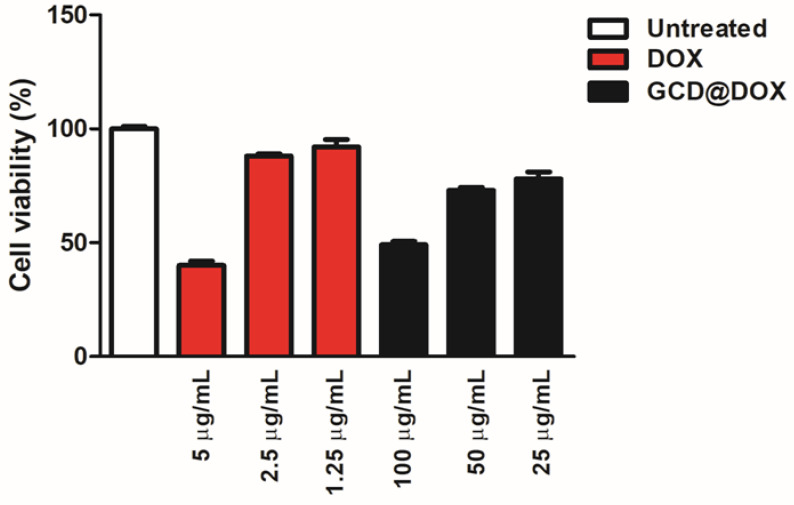
In vitro study of the biocompatibility of GCD@DOX in HEp-2 cells. The data showed the % of live cells compared to the control (free DOX at 5, 2.5 and 1.25 μg/mL) and cells only after 24 h. Cells were exposed to 100, 50 and 25 μg/mL of GCD@DOX corresponding to 2.5, 1.25 and 0.625 μg/mL of loaded DOX, respectively. The cellular proliferation index (%) was determined on the basis of ATP level using ViaLight™ plus cell proliferation and cytotoxicity bioassay kit (Lonza Group Ltd., Basel, Switzerland) in combination with GloMax Multi Microplate Luminometer, as described in Materials and Methods. The GraphPad Prism 6 software was used for data analysis and for graphical representation. The assay was performed as means of triplicates ± SD.

Cytotoxicity of GCD in different cell lines was investigated in our previous study [16]. Specifically, in tumor HEp-2 cells a cellular proliferation percentage of 75, 80 and 100% was detected after their exposition to graphene at 100, 50 and 25 μg/mL, respectively [16]. To complete the investigations on the cytotoxic effect of the whole drug delivery system (GCD@DOX), additional experiments (Figure 6) were performed by using free DOX (5, 2.5 and 1.25 μg/mL) and untreated HEp-2 cells as positive and negative controls, respectively. The cells were treated for 24 h with GCD@DOX at the concentrations of 100, 50 and 25 μg/mL corresponding to nominal DOX concentration of 2.5, 1.25 and 0.625 μg/mL, respectively. The results graphically represented as percentage of cell viability (Figure 6) showed a decrease of 40% and 76% of cellular proliferation after exposition to 100 and 50 μg/mL of GCD@DOX (DOX loading corresponding to 2.5 and 1.25 μg/mL, respectively). These biological outcomes were comparable with the effects of free DOX at 5 and 2.5 μg/mL that showed 40% and 88% of cellular proliferation (Figure 6).

### 2.4. Gene Expression Modulation in HEp-2 Cells

**Figure 7:**
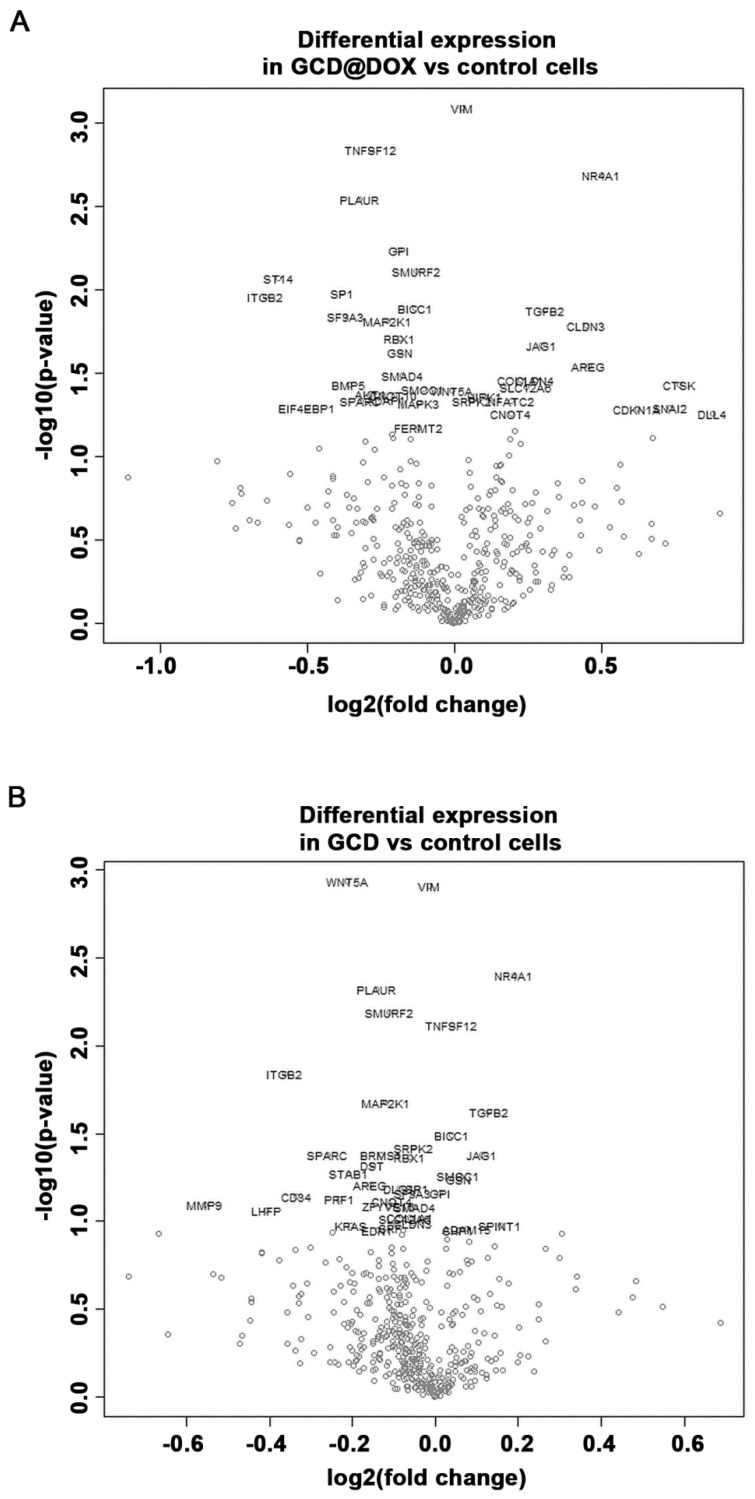
Volcano plot showing changes in the gene expression profile of cells treated with GCD@DOX (**A**) and GCD (**B**) in comparison to control cells. Genes with statistically significant differential expression fall at the top of the plot, and highly differentially expressed genes fall to either side depending on whether they are negatively or positively differentially expressed.

The cancer transcriptome from control and treated HEp-2 cells was profiled using the NanoString nCounter™ platform. We evaluated changes in the expression of genes involved in processes associated to angiogenesis, extracellular matrix modification (ECM) and tumor metastasis induced by GCD and GCD@DOX in HEp-2 cells at sub-toxic concentration (25 μg/mL). Although the gene expression profile in control and treated cells was similar (Figure 7), the analysis revealed a differential expression of 34 genes, 21 of which were down-regulated and 13 were up-regulated, in cells treated with GCD@DOX in comparison to control cells (Table 2). In particular, genes encoding for the proteins, such as Matriptase (*ST14*), Integrin-beta2 (*ITGB2*), Jagged 1 (*JAG1*), Amphiregulin (*AREG*), Cathepsin K (*CTSK*), and Osteonectin (*SPARC*) were expressed at very low levels as evidenced by the low raw count (below 100). Notably, all of these genes are related to tumorigenicity and tumor invasiveness (Figure 7).

Among the highly expressed transcripts, the genes encoding for Gelsolin (*GSN*), Bone morphogenetic protein 5 (*BMP5*) and Serine/Threonine Kinase 1 (*AKT1*) related to angiogenesis, ECM and metastasis were down-regulated in GCD@DOX treated cells. At the same time, the transcript levels of genes encoding for claudin-3 (*CLDN3*), the nuclear receptor subfamily 4 group A member 1 (*NR4A1*), and the transforming growth factor β (*TGFB2*) related to angiogenesis, hypoxic response and tumor invasion were up-regulated.

Regarding the impact of GCD on cancer cell biology, the results evidenced that also in cells treated with GCD alone the expression of 16 genes was significantly regulated in comparison to controls, 11 of these genes were down-regulated and five were up-regulated (Figure 7). As shown in Table 2, 14 common genes were differentially expressed in GCD@DOX or GCD treated cells in comparison to controls. Of note, four of these genes encoding for vimentin (*VIM*), Tumor Necrosis Factor (Ligand) Superfamily Member 12 (*TNFSF12*), BicC RNA Binding Protein 1 (*BICC1*) and Serine/Arginine-Rich Protein-Specific Kinase 2 (*SRPK2*) had an opposite trend in the two treatments. Indeed, the transcript levels of TNFSF12 and BICC1 were increased by GCD exposure (0.0395 and 0.0386 log2 fold, respectively) and reduced by GCD@DOX treatment (−0.281 and −0.132 log2 fold, respectively). While, the expression of VIM and SRPK2 was down-regulated in cells treated with GCD (−0.0134 and −0.0541 log2 fold, respectively) and up-regulated in cells treated with GCD@DOX (0.0277 and log2 fold, respectively) (Table 2).

However, in comparison to control cells, the changes induced by treatment with GCD were smaller than those observed in cells treated with GCD@DOX (see Table 2). For example, the expression of NR4A1 was increased by 0.5 log2 fold in cell treated with GCD@DOX when compared to control cells, while the GCD treatment induced an increase of only 0.19 log2 fold in comparison to controls. Similarly, the expression of TGFβ2 was up-regulated by 0.309 log2 fold in cells treated with GCD@DOX, and by 0.129 log2 fold in cell treated with GCD in comparison to control cells (Table 2).

## 3. Discussion

In this study, we investigated the cellular fate of a new drug delivery system based on graphene cationic cyclodextrin platform entrapping DOX (GCD@DOX) and the modulation of expression of some genes related to cancer progression after the cell exposition on graphene materials (GCD and GCD@DOX). The cellular fate of GCD@DOX was tracked by complementary techniques: FLIM and Raman mapping investigations emphasized the localization of GCD@DOX complex only in the cytoplasm (Figure 3 and Figure 4) mainly located in the proximity of the nucleus; whereas microscopy fluorescence visualized DOX mainly in the nucleus (Figure 5). As a whole, our experiments proved an efficient cellular uptake of GCD@DOX together with the presence of DOX in the nucleus without graphene carrier. These findings suggested the release of DOX from GCD@DOX platform adjacent to perinuclear region and the subsequent nuclear internalization and widespread in the nuclear compartment. We investigated the cellular fate of GCD@DOX by complementary spectroscopic techniques applied directly on murine C26 cell line or on human HEp-2 cell line. Some drawbacks, such as HEp-2 death events during Raman mapping experiments and the indistinguishable lifetime signal of free DOX in the cell, hampered the direct tracking of GCD@DOX or its components in human cell lines by FLIM and Raman mapping.

Cytotoxicity results (Figure 6) evidenced a higher impact on the cell viability of HEp-2 tumor cells treated with encapsulated DOX respect to ones treated with free drug at the same concentration. Specifically, a comparable biological outcome (40% of cellular proliferation) was found for loaded DOX 2.5 μg/mL (100 μg/mL of GCD@DOX) and free DOX at 5 μg/mL. This effect could be ascribed to the peculiar caveolae-mediated endocytosis internalization pathway [16] of GCD that could enhance the GCD@DOX concentration in the perinuclear region and/or to the synergic effect of graphene (GCD) on the modulation of gene expression of DOX. From our study, it clearly emerged that tuning of gene expression profile was induced by GCD or DOX loaded on graphene (GCD@DOX), even if both were investigated at sub-toxic concentration. The HEp-2 cells were treated with GCD@DOX (25 μg/mL) or GCD (25 μg/mL) that produced no significant cytotoxicity (cell proliferation of 80% and 100%, respectively).

Literature data on the gene expression analysis after the exposure to graphene materials is very limited, and the attention was especially devoted to the impact on the gene involved in immune responses [18]. Interestingly, graphene oxide (GO) at sub-toxic concentrations has been reported to compromise plasma membrane and cytoskeleton in J774A.1 macrophages and A549 lung cancer cells, with no significant cytotoxic effects. Moreover, the interactions of GO with integrins activated the integrin–FAK–Rho–ROCK pathway, leading to suppression of integrin expression as well as perturbation of cell membrane and cytoskeleton, and a subsequent cellular priming state [28]. The polyethylene glycol-diamine/R8-functionalized GO has been shown to effectively down-regulate c-Myc protein and EGFP expression [29]. The treatment with GO-poly-l-lysine hydrobromide/folic acid (GPF)/DOX/siRNA was able to silence gene expression and inhibit tumors by about 70% without exhibiting testable cytotoxicity; moreover, there was a decrease of 50% in vascular endothelial growth factor (*VEGF*), mRNA, and protein levels [30]. GO nanohybrids, used for the delivery of chemotherapeutic agent fluorouracil (FU) against breast cancer MCF7 cells, increased the protein levels of p53 and cleaved PARP proteins [31].

In this paper, we pointed out the importance of looking at the genomic level by using large genome expression analysis technologies to better understand the impact of graphene materials on cancer cell biology. Our study revealed that GCD@DOX was able to change the expression of some genes related to cancer progression, and in most cases, a convergent effect of GCD was detected. It is important to point out that the cellular response for both materials was observed at concentration that do not elicit acute cytotoxicity.

In particular, GCD@DOX treatment induced a strong down-regulation of some genes, including *ST14*, *PLAUR*, *SP1*, *SF3A3*, *ITGB2*, *GSN*, *BMP5*, *TNFSF12*, and *AKT1*, which are involved in the regulation of the multistep process of cancer development, invasion and metastasis. The modulation of these genes, except *TNFSF12*, *ITGB2*, and *PLAUR*, is due to DOX effect since no changes were detected in the cells treated with GCD alone.

Contemporarily, GCD@DOX treatment caused the increase in the expression of *CLDN3*, *NR4A1*, and *TGFB2* genes, which may also affect cell response to hypoxic stimuli and epithelial to mesenchymal transition (EMT). The effect of GCD on *NR4A1* and *TGFB2* is converging to DOX action. Matriptase, encoded by *ST14* (suppressor of tumorigenicity 14 protein), is known to cleave and activate urokinase plasminogen activator, and it has been associated with cancer invasion and metastasis of many tumor types [32]. The urokinase plasminogen activator receptor (uPAR), encoded by Plasminogen Activator, Urokinase Receptor (PLAUR), has been involved in angiogenesis, growth, and metastasis of many hematologic and solid tumors, and has recently been shown as a prognostic factor for oral squamous cell carcinoma [33,34]. *SP1* encodes the transcription factor (TF) Sp1, that has been associated with poor prognosis for various tumors; moreover, functional studies demonstrated that Sp TFs regulate the expression of genes responsible for cancer cell growth, survival, migration/invasion, inflammation, and drug resistance [35]. *SF3A3* gene is a component of the spliceosome that represents a useful therapeutic target in non-small cell lung cancer, since its silencing is associated with induction of cell cycle arrest and cell death [36].

Gelsolin *(GSN*) is one of the most abundant actin-binding protein modulating cell motility, shape and metabolism. Moreover, it participates in immunologic processes through interactions with different cells of the immune system, and has been recognized as a potential biomarker of inflammation-related pathological conditions. GSN over expression has been associated with the progression of oral carcinoma. In addition, several reports indicated GSN as a protein affecting human colon cancer and melanoma cell motility. A very recent report demonstrated that GSN is able to inhibit the malignant phenotype of glioblastoma [37,38,39]. Because of its important role in metastatic processes, as well as in different cellular mechanisms and cellular interactions, GSN has been suggested as a potential target for various therapeutic treatments [40].

RAC-alpha serine/threonine-protein kinase 1 (*AKT1*) is also known to promote metastasis, but several data reported its ability to inhibit cancer cell invasion [41]. Literature studies revealed opposite activity of AKT1 in the modulation of cell migration in cells with different origin. For example, a study on human breast cancer cell lines showed that AKT1 inhibits cell migration and invasion [42], while using mouse embryonic fibroblasts AKT1 has been found to promote migration [43]. AKT up-regulation and activation have been associated with increased cellular content of phosphatidyl-inositol-3-phosphate (PIP3) and phosphatidyl-inositol-3-kinase activity as well as with increased mRNA and protein amounts of Twist, a transcription factor promoting EMT. Notably, these effects are inhibited by claudin 3 (*CLDN3*) and claudin 4 up-regulation [44].

In our work, *AKT1* down-regulation was associated with claudin 3 *(CLDN3)* up-regulation, thus confirming the aforementioned observations.

CLDN3 belongs to a family of integral membrane proteins forming the backbone of tight junction. Alterations of *CLDN3* gene expression have been frequently observed in several human cancers, with discordant findings about their role [45]. *CLDN3* over-expression has been reported in all subtypes of epithelial ovarian cancers, and appeared to enhance angiogenic and invasive properties [46]. Also, in lung adenocarcinoma *CLDN3* overexpression resulted in promoting the malignant potential [47]. Conversely, *CLDN3* downregulation, frequently reported in human liver cancer, was associated with a worse prognosis of patients with hepatocellular carcinoma [48]. CLDN3 functions to sustain an epithelial phenotype and its loss promotes EMT [44].

Conflicting results have also been evidenced for bone morphogenetic protein 5 (*BMP5*), a member of the TGFβ family. The loss of BMP5 seems to be an early event in CRC initiation and development, on the contrary a significant increase of BMP5 positive rate has been found in breast carcinoma in comparison to normal tissues suggesting that the role of BMP5 varies among tumors [49].

As mentioned above, GCD alone was able to significantly perturbate the expression of some genes, inducing the down-regulation of and *TNFSF12*, and the up-regulation of *NR4A1* and *TGFβ2*. These effects were synergistically reinforced by GCD@DOX.

ITGB2 (Integrin beta-2), also known as leukocyte-specific CD18, plays a key role in cell adhesion and cell surface signaling, as well as in immune response. ITGB2 expression in cancer cells promotes cell invasion through the endothelium in leukocyte-like manner. *ITGB2* levels are significantly increased in metastatic prostate cancer [50]. Notably, the observed GCD-induced down-regulation of *ITGB2* confirms previously reported effects of graphene on integrin suppression linked to plasma membrane and cytoskeleton meshwork perturbation [51].

Tumor necrosis factor ligand superfamily member 12 (*TNFSF12*) is a member of the tumor necrosis factor superfamily that is also known as TNF-related weak inducer of apoptosis (TWEAK). TNFSF12 acts through the binding to the receptor Fn14 (fibroblast growth factor-inducible 14), which in turn triggers the activation of several intracellular signal transduction downstream pathways, also including those involving tumor necrosis factor, receptor-associated factor and nuclear factor kappa B, leading to survival, proliferation, migration, or cell death depending on cell microenvironment. Indeed, the activation of TWEAK/Fn14 signaling pathways has been shown to promote angiogenesis, proliferation, EMT, invasion, and migration of tumor cells. Moreover, TWEAK up-regulated expression has been reported in many solid tumors compared with healthy tissues [51].

A controversial role has been reported for the nuclear receptor subfamily 4 group A member 1 (NR4A1), and transforming growth factor beta 2 (TGFβ2), whose gene expression resulted increased by GCD@DOX treatment in our study. The role of NR4A1 protein in cancer is also currently unclear. NR4A1 has been found overexpressed in lung, pancreatic, and colon carcinoma, exhibiting a tumor-promoting effect [52,53]. The up-regulation of NR4A1, detected in both ER-positive and negative breast cancer, was correlated with a decreased relapse-free survival in breast cancer [54]. Conversely, in human triple-negative breast cancer the NR4A1 expression was negatively correlated with tumor stage, lymph node metastasis and disease recurrence, and in mouse models of basal-like mammary tumors a progressive NR4A1 reduction has been found during cancer development (Nuclear receptor NR4A1 is a tumor suppressor down-regulated in triple-negative breast cancer).

Members of the TGF-beta family control physiological processes, such as angiogenesis, cell proliferation, differentiation, adhesion and migration. In particular, it has been hypothesized that TGFβ2 may have different effects on tumor growth at different stages of the disease, acting as an inhibitor at the early stages and as activator at the late stages [55,56]; moreover, it has recently been shown to antagonize IL-6-induced survival and promote cell apoptosis [57].

The increase of *VIM* expression is correlated to EMT, an event that has been shown to be associated with resistance and poor outcome treatment in patients with different tumors [58]. Interestingly, our investigation showed a divergent effect of DOX and GCD on *VIM* expression; specifically, the detrimental increase of *VIM* expression induced by DOX was softened by GCD-induced *VIM* down regulation.

GCD@DOX treatment also reversed the down regulating effects of GCD alone on *SRPK2* gene. An oncogenic role of SRPK1/2 (Serine/arginine-protein kinase) was reported in various types of tumors, including leukemia and melanoma as well as pancreatic, breast, colon, lung, and ovarian cancer [59], hence different strategies have been proposed for the *SRPK1/2* suppression [60]. In our opinion, the down-regulation of *SRPK2* induced by GCD, even if weak, is noteworthy since this GCD ability may be exploited in the development of SRPK1/2 inhibitor delivery systems in which the graphene can act in synergy with specific SRPK1/2 inhibitors.

## 4. Materials and Methods

### 4.1. Cell Culture

The in vitro study of intracellular uptake and the NanoString^®^ Analysis were performed on HEp-2 (American Type Culture Collection, ATCC, P.O. Box 1549, Manassas, VA 20108, USA).

The cells were grown in RPMI 1640 medium (Lonza, Group Ltd Muenchensteinerstrasse 38 CH-4002 Basel, Switzerland), supplemented with 10% FBS, 100 U/mL penicillin and 100 μg/mL streptomycin mixture and were cultured at 37 °C in a 5% CO_2_ incubator. Murine colon carcinoma cells (C26, Cell Line Service, Germany) were grown in RPMI culture medium (Lonza), supplemented with 2 mM L-glutamine, penicillin/streptomycin 100 U/mL, 10% fetal calf serum and incubated in a humidified incubator (37 °C, 5% CO_2_). For Raman imaging, 3 × 10^4^ cells/dish were grown on Ibidi μ-Dishes (ibidi GmbH Gräfelfing, Germany) with glass bottoms (35 mm, high wall, uncoated). For FLIM assay, 2 × 10^5^ cells/dish were seeded on Ibidi μ-Dish (50 mm, low wall, ibiTreat coating). After 24 h of cultivation, the culture medium was replaced with complete culture medium containing GCD@DOX (15 μg/mL) and incubated for additional 24 h. After incubation, the cells were rinsed with phosphate buffered saline (PBS) to remove the non-internalized GCD@DOX platforms. Untreated cells were used as a control sample.

### 4.2. Cell Viability Assay

The cell viability of HEp-2 cells treated with GCD@DOX and DOX was determined on the basis of ATP levels using ViaLightTM plus cell proliferation and cytotoxicity bioassay kit according to the manufacturer’s instructions (Lonza Group Ltd., Basel, Switzerland). Cells were grown in wells of 96-well plates and treated with different concentrations of GCD@DOX (100 μg/mL, 50 μg/mL and 25 μg/mL) or free DOX (5 μg/mL, 2.5 μg/mL and 1.25 μg/mL). After the indicated incubation time the cells were harvested and the emitted light intensity related to ATP degradation was quantified with the GloMax Multi Microplate Luminometer (Promega Corporation, WI, USA). The luminescence value was converted to the cell proliferation index (%) according to the following equation:

(1) Cell viabillity%=[(A−B)/ (C−B)]%

where A denotes the average luminescence of treated samples, B represents background luminescence and C represents the average of untreated samples.

### 4.3. Intracellular Uptake of GCD@DOX

HEp-2 cells were untreated or treated with GCD@DOX (25 μg/mL) for 24 h. DOX was used as a control (1.25 μg/mL). The samples were collected and washed with warm PBS for twice, fixed with PFA 4% for 30 min at room temperature and permeabilized with Triton 0.1% for 1 h, covered with a drop of mounting solution (ProLong™ Diamond Antifade Mountant with DAPI-Invitrogen p36971) for 30 min in a dark room. The images were captured and processed using confocal laser scanning microscopy (TCS SP8, Leica, (TCS SP8, Leica Microsystems Srl, Milan, Italy); magnification, 63×.

### 4.4. Characterization Techniques

Dynamic light scattering (DLS) and ζ-potential measurements were carried out according our previously reported protocols [16].

A confocal Raman microscope (CRM alpha 300R from WITec GmbH, Ulm, Germany) was employed to perform the Raman measurements. The Raman measurements on dried droplets of GCD and GCD@DOX were conducted at a power incident on the samples of 1 mW and the signal was collected through a 100× objective (NA = 0.9) with an integration time of 10 s for each spectrum. The 532 nm line of a Nd-YAG was used as excitation. The reference Raman spectrum of DOX powder was recorded by using an excitation line at 785 nm. The Raman maps of the live C26 colon carcinoma cells were recorded on the same WITec confocal Raman microscope using the 532 nm line as excitation wavelength. The Raman signal collected through a W-plan Apochromat 63× water immersion objective (NA = 1, WD = 2.1 mm, Zeiss, Munich, Germany) was passed through a holographic edge filter and then focused into a multimode optical fiber of 100 µm diameter which provides the optical pinhole for confocal measurement. The light emerging from the output optical fiber was analyzed by an ultrahigh throughput spectrometer equipped with a back-illuminated deep-depletion 1024 × 128-pixel CCD camera operating at -60 °C. The measurements were conducted by choosing an integration time of 0.5 s for each spectrum. The WITec ProjectPlus software was used for spectral analysis and image processing. FLIM was performed on a PicoQuant MicroTime 200 (Picoquant GmbH, Berlin Germany) time-resolved inverted confocal fluorescence microscopy (IX 71, Olympus, Tokyo, Japan) equipped with a Plan N 40×/numerical amperture (NA) = 0.65 objective. The excitation beam was provided via a fiber-coupled, picosecond diode laser head (LDH-D-C 485, 0.55 μW, PicoQuant) operating at 485 nm (40 MHz). The signal collected through the objective was spatially and spectrally filtered by a 50 µm diameter confocal pinhole, long-pass emission filter (HQ485LP, Chroma Technology, Brattleboto, Bellows Falls, VT, USA) and a photon counting detector module (PDM series, Microphoton devices), connected to a time-correlated single photon counting (TCSPC) module (PicoHarp 300, PicoQuant, Germany).

All images and time-fluorescence decay curves were acquired and analyzed using the SymPhoTime software (version 1.6) provided by PicoQuat. For FLIM image acquisition was employed a piezo x−y-scanning table and a PiFoc z-piezo actuator for microscope objective. In vitro fluorescence lifetimes of free DOX and DOX loaded onto the GCD (GCD@DOX) were firstly measured in solution, and subsequently their internalization into the C26 colon cells were monitored using the FLIM system well-described above. The spectral response of the GCD@DOX therapeutic platform internalized inside the cells were also investigated by employing an SR-163 spectrograph equipped with a Newton 970 EMCCD camera from Andor Technology (Belfast, UK), which was attached to an exit port of the MicroTime 200 optical unit. The integration time used for the acquisition of the emission spectrum was 60 s. Bright field image of the GCD@DOX treated C26 colon cells scanned by FLIM was acquired using an Olympus CAM-XC30 digital camera with active Peltier cooling using the same microscope and an Olympus IX-2 LW UCD (NA = 0.55) condenser.

### 4.5. Preparation of GCD@DOX

GCD@DOX complex was prepared by mixing 3 mL of GCD dispersion (1 mg/mL) and 1.4 mL of DOX solution (0.12 mg/mL); the dispersion was sonicated for 20 min by a microtip probe, under bath ice (UW 2070 SONOPLUS, Bandelin Electronic, (Berlin, Germany) and then it was centrifuged at 5000 RPM for 15 min. The precipitate was washed twice with superpure water up to obtain a colorless supernatant which was removed. The collected supernatants were analyzed by UV-Vis at 477 nm absorbance maximum (ε_Dox (477 nm)_ ≈ 11,500 cm^−1^ M^−1^) to estimate the excess of DOX and determine the actual DOX loading in the complex. The precipitate was lyophilized before the use (recovery yield of ≈ 40%). The actual DOX loading was of 2.5% and the entrapment efficiency percentage (EE%) was >95%.

### 4.6. RNA Isolation and NanoString^®^ Analysis

Total RNA was extracted from HEp-2 cells treated with GCD@DOX (25 μg/mL), GCD (25 μg/mL) and control cells (in duplicate) using GenElute™ Mammalian Total RNA Miniprep Kit (Sigma Aldrich, Milan, Italy) according to the manufacturer’s instructions. Total RNA was quantified with Thermo Scientific™ NanoDrop™ 8000 (Thermo Scientific, Monza, Italy) and stored at −80 °C until expression testing.

RNA transcript amounts were analyzed using NanoString^®^ technology, which allows for direct multiplexed measurements of gene expression from a low amount of mRNA (25–300 ng) without the need for cDNA synthesis or PCR. The RNA nCounter PanCancer Human Progression Panel, which includes 770 genes (angiogenesis, cancer metabolism, extracellular matrix remodeling (ECM), epithelial-to-mesenchymal transition (EMT), hypoxia, metastasis, tumor growth, tumor invasion and 30 reference genes) was used. Briefly, RNA (100 ng) was hybridized for 16 h using capture and reporter probes according to the manufacturer’s protocol. Then, the samples were hybridized into the cartridges using the nCounter Prep Station and digital images from prepped cartridges were processed within the Nanostring Digital Analyzer. Analysis of read-count data including imaging QC, read-count QC, and differential expression was performed using the nSolver™ 3.0 with Nanostring Advanced Analysis Module 2.0 plugin, according to the guidance given by the manufacturer. After applying a negative control subtraction, raw counts were first normalized with internal controls (Nanostring POS controls) and with the expression of ten housekeeping genes present in the panel, and then Log2 transformation was applied to the expression matrix. NanoString data were processed in the R statistical environment.

## 5. Conclusions

This work investigates the intracellular trafficking in C26 and HEp-2 cells of graphene functionalized with cationic cyclodextrin (GCD) loaded with the anticancer agent doxorubicin (DOX) and the related modulation of the expression of some genes involved in cancer biology. The cellular fate of GCD@DOX was tracked by complementary techniques including FLIM, Raman mapping and fluorescence microscopy.

Biological outcomes evidenced the efficient cellular uptake of GCD@DOX and the presence of DOX in the nucleus without graphene carrier. These findings suggested the release of DOX from GCD@DOX platform close to perinuclear region and the subsequent nuclear internalization and widespread in the nuclear compartment. Moreover, cytotoxicity studies showed a higher effect on the cell viability of tumor HEp-2 cells treated with encapsulated DOX respect to HEp-2 cells treated with free drug at the same concentration. Probably, the peculiar caveolae-mediated endocytosis internalization pathway of GCD platform could increase the delivery of DOX into the nucleus, optimizing its biological action.

Overall, NanoString nCounter™ platform, a novel digital molecular barcoding technology, provided evidence for 34 (out of 700) differentially expressed cancer-related genes in cells treated with GCD@DOX compared with untreated cells (16 in cells treated with GCD alone). Given the different roles in tumor biology of the aforementioned proteins and considering the small number of genes significantly modified by GCD@DOX treatment, it is not possible to identify a distinct molecular pathway activated or inhibited by GCD@DOX. Therefore, the present findings are not conclusive of GCD@DOX effectiveness against HEp-2 cell growth and invasiveness, and are worthy of further studies. However, data obtained by gene expression analysis provide useful information on the impact of graphene materials on cancer cell biology and may guide further efforts towards effective graphene-based drug delivery systems. This study unveiled the ability of GCD to sensitize cancer cells to chemotherapeutic drugs (i.e., DOX); therefore, chemotherapy efficacy can be enhanced by GCD co-treatment. Finally, we point out that the positive or negative effects related to down/up-regulation of genes involved in cancer metastasis and progression were detected at doses that do not elicit acute cytotoxicity. Further investigations will be performed to obtain information about the regulation of gene expression in response to changing the time of exposure and the concentration of the drug delivery system.

## Figures and Tables

**Table 1:** Overall Properties of GCD and GCD@DOX in water.

Sample	D_H_ (µm ± SD) ^1,2^ (%) ^3^	PDI	ζ (mV ± SD)	Theoretical Loading (%)	^3^ Actual Loading (%)	^4^ EE (%)
GCD	>1 μm	≥ 0.4	−9.5 ± 0.3			
GCD@DOX	1 μm ± 0.2	≥ 0.4	−16.6 ± 4	2.6	2.5	95

^1^ SD was calculated on three different batches. ^2^ Size with corresponding intensity % distribution. ^3^ Actual loading is expressed as the amount of drug (mg) encapsulated per 100 mg of complex. ^4^ Ratio between actual and theoretical loading ×100.

**Table 2:** List of differentially regulated genes in HEp-2 cells treated with GCD@DOX or GCD in comparison with control cells.

	GCD@DOX vs. Ctrl ^3^		GCD vs. Ctrl	
Gene	Log2 Fold Change	*p*-Value	Log2 Fold Change	*p*-Value
VIM ^1^	0.0277	0.000813	−0.0134	0.00124
TNFSF12 ^1^	−0.281	0.00145	0.0395	0.00759
NR4A1	0.5	0.00206	0.19	0.004
PLAUR	−0.322	0.00286	−0.142	0.00477
GPI	−0.187	0.0058		
SMURF2	−0.127	0.00774	−0.112	0.00649
**ST14** ^2^	−0.597	0.00851		
SP1	−0.379	0.0105		
**ITGB2** ^2^	−0.641	0.011	−0.362	0.0144
BICC1 ^1^	−0.132	0.0128	0.0386	0.0323
TGFβ2	0.309	0.0134	0.129	0.0238
SF3A3	−0.368	0.0144		
MAP2K1	−0.225	0.0153	−0.12	0.0212
CLDN3	0.45	0.0164		
RBX1	−0.185	0.0194	−0.0617	0.0429
**JAG1** *^2^*	0.298	0.0214	0.113	0.0416
GSN	−0.184	0.0238		
**AREG** ^2^	0.456	0.0285		
SMAD4	−0.174	0.0327		
COL1A1	0.226	0.0345		
CLDN4	0.278	0.0349		
**CTSK** ^2^	0.766	0.037		
BMP5	−0.358	0.0373		
SLC12A6	0.246	0.0385		
SMOC1	−0.106	0.0394		
WNT5A	−0.00843	0.0398	−0.211	0.00117
AKT1	−0.283	0.0423		
CNOT10	−0.202	0.0428		
HIPK1	0.103	0.0438		
ADAP1	−0.23	0.0455		
SRPK2 ^1^	0.0604	0.0464	−0.0541	0.0382
**SPARC** ^2^	−0.319	0.0464	−0.259	0.0421
NFATC2	−0.194	0.0466		
MAPK3	−0.122	0.0481		
BRMS1			−0.13	0.0416
DST			−0.152	0.0481

^1^ Gene with an opposite trend in cells treated with GCD@DOX and GCD. ^2^ Genes with raw count lower than 100 are shown in bold. ^3^ HEp-2 not treated cells are used as control.

## Abbreviations

G	Graphene
GCD	Graphene modified with cationic cyclodextrins
DOX	Doxorubicin
GCD@DOX	Graphene cationic cyclodextrin platform entrapping doxorubicin
DLS	Dynamic light scattering
FLIM	Fluorescence lifetime imaging microscopy
TEM	Transmission electron microscope
HEp-2 cells	Human epithelial type 2 cells
ECM	Extracellular matrix
ST14	Matriptase
ITGB2	Integrin-beta2
JAG1	Jagged 1
AREG	Amphiregulin
CTSK	Cathepsin K
SPARC	Osteonectin
GSN	Gelsolin
BMP5	Bone morphogenetic protein 5
AKT1	Serine/Threonine Kinase 1
CLDN3	Claudin-3
NR4A1	Nuclear receptor subfamily 4 group A member 1
TGFβ2	Transforming growth factor β
VIM	Vimentin
TNFSF12	Tumor Necrosis Factor (Ligand) Superfamily Member 12
BICC1	BicC RNA Binding Protein 1
SRPK2	Serine/Arginine-Rich Protein-Specific Kinase 2
